# Role of Host Small GTPases in Apicomplexan Parasite Infection

**DOI:** 10.3390/microorganisms10071370

**Published:** 2022-07-07

**Authors:** Silvio Paone, Anna Olivieri

**Affiliations:** 1 Department of Infectious Diseases, Istituto Superiore di Sanità, 00161 Rome, Italy; silvio.paone@uniroma1.it; 2 Department of Public Health and Infectious Diseases, Sapienza Università di Roma, 00161 Rome, Italy

**Keywords:** Apicomplexa, GTPases, malaria, *Plasmodium*, *Toxoplasma*, *Cryptosporidium*, *Theileria*, host–parasite interactions, host-targeted therapies

## Abstract

The Apicomplexa are obligate intracellular parasites responsible for several important human diseases. These protozoan organisms have evolved several strategies to modify the host cell environment to create a favorable niche for their survival. The host cytoskeleton is widely manipulated during all phases of apicomplexan intracellular infection. Moreover, the localization and organization of host organelles are altered in order to scavenge nutrients from the host. Small GTPases are a class of proteins widely involved in intracellular pathways governing different processes, from cytoskeletal and organelle organization to gene transcription and intracellular trafficking. These proteins are already known to be involved in infection by several intracellular pathogens, including viruses, bacteria and protozoan parasites. In this review, we recapitulate the mechanisms by which apicomplexan parasites manipulate the host cell during infection, focusing on the role of host small GTPases. We also discuss the possibility of considering small GTPases as potential targets for the development of novel host-targeted therapies against apicomplexan infections.

## 1. Introduction

Apicomplexa are a large monophyletic phylum of Protista, belonging to the group *Alveolata*. It comprises more than 6000 species, most of which are obligate intracellular parasites, including species responsible for important human diseases, such as malaria, cryptosporidiosis and toxoplasmosis. These three diseases are a major threat to human health. In 2020, 241 million people contracted malaria and 627,000 died, mainly in Africa, and 77% of the deaths were reported in children under five years of age [1]. Malaria causative agents, *Plasmodia*, are transmitted by *Anopheles* mosquitoes to a vertebrate host, where they undergo a first replicative cycle in hepatocytes followed by continuous cycles of infection in erythrocytes.

*Toxoplasma gondii*, the etiological agent of toxoplasmosis, infects one-third of the global population, with the highest rates in Africa and South America, causing abortion or severe birth defects if transmitted to the fetus during pregnancy by seronegative woman [2]. Moreover, in immunocompromised individuals, toxoplasmosis is one of the main opportunistic infections and can cause several clinical manifestations such as encephalitis, chorioretinitis and pneumonitis or disseminated infection and even death, depending upon the immune status of the host [3]. *T. gondii* invades and replicates within most nucleated cells of warm-blooded animals. It is transmitted to humans through consumption of raw or undercooked meat of intermediate hosts or by ingestion of oocysts excreted by domestic cats or other members of the family *Felidae*.

*Cryptosporidium parvum* infects mainly epithelial cells in the intestine after ingestion of oocysts from contaminated food or water or through direct contact with an infected person or animal. The main symptoms of infection are diarrhea and abdominal cramps and, in patients with a weakened immune system, symptoms can be very severe, causing about 50,000 deaths worldwide every year [4].

The life cycles of apicomplexan parasites are complex, alternating between asexual and sexual replication and often involving infection of two or more different hosts. A common characteristic of apicomplexan parasites is their “apical complex”, formed by specialized cytoskeletal structures and secretory organelles localized at the parasite apical end. These secretory organelles, called micronemes, rhoptries and dense granules, release their content in a sequential fashion upon interaction with the host cell, mediating attachment and invasion. Immediately after attachment, parasite and host cell plasma membranes are tightly juxtaposed, forming the so-called *moving junction* (MJ), a ring-like structure formed by microneme and rhoptry proteins through which parasites slide to penetrate inside the host cell. This process is sustained by an active form of motility typical of apicomplexans, named “gliding”, which depends on the retrograde translocation of surface-exposed microneme proteins by the actinomyosin motor of the parasite. Together, apical complex and gliding motility ensure that apicomplexan parasites have a characteristic, active and fast mechanism of host cell invasion, strictly dependent on finely tuned parasite–host cell interactions [5]. After invasion, most apicomplexan parasites reside inside a parasitophorous vacuole (PV), a compartment formed by an invagination of the host cell membrane.

The long parasitic behavior has led apicomplexan parasites to develop strategies in order to survive in so many different conditions [6]. As an example, these parasites have lost the ability to synthesize several amino acids and evolved different transmembrane transporters in order to scavenge them from the host cell [7]. Moreover, they have also lost the ability to synthetize some lipid classes. Indeed, *T. gondii* and *Plasmodium falciparum*, the most lethal among human malaria parasites, are both auxotrophic for cholesterol and need to acquire it from the host. Apicomplexa also scavenge sphingolipids, fatty acids and phospholipids from the host and produce their own lipids through a relic plastid called apicoplast. Upon acquisition from the host, the acquired lipids are often assembled together with parasite lipids in order to generate most lipid species important for parasite needs [8].

Apicomplexa also scavenge nutrients from the host cell [9] by subverting the host cell trafficking of nutrient-loaded vesicles and the subcellular localization of organelles involved in vesicle maturation and nutrients transport, such as the Golgi network and the endoplasmic reticulum [10,11,12,13,14,15,16,17].

Moreover, apicomplexan parasites widely hijack different host cell factors during their intracellular life, manipulating several aspects of cell functioning, to create a favorable niche for their development inside the host [18,19].

The modification of the host cell cytoskeleton is key to apicomplexan parasite survival, both during host cell invasion and the subsequent intracellular development. This manipulation is achieved either by a direct interaction between the parasite and the host cytoskeleton or by hijacking host signaling pathways involved in cytoskeleton regulation [13,20].

## 2. Manipulation of the Host Cell Cytoskeleton in Apicomplexan Parasite Infection

The cytoskeleton is a major cellular component that provides structural support and assists with numerous cellular functions. It consists of three polymeric networks: microfilaments, formed by actin; microtubules, formed by tubulin; and intermediate filaments, formed by several different proteins, including the widely distributed filament protein vimentin.

Invasion of the host cell is a critical step for parasite survival, since it requires a series of coordinated events to occur in sequence in a short time. This process is widely mediated by the manipulation of the cytoskeleton that lies beneath the host plasma membrane [21].

During invasion of *T. gondii* parasites, host microtubules early localize at the moving junction and treatment of host cells with nocodazole, a drug that disrupts microtubules, reduces invasion, indicating that they play a role in this process, possibly providing the parasite with an anchor point during host cell penetration [22]. Consistent with this, it was also shown that during invasion *T. gondii* exports a protein called rhoptry neck protein 4 (TgRON4) to the host cytoplasmic side of the moving junction, where the protein directly binds to the host microtubules [23].

During invasion, both *T. gondii* and *Plasmodium berghei*, the causative agent of malaria in rodents, induce the formation of a host-derived F-actin structure in proximity to the MJ. Pre-treatment of host cells with the actin-destabilizing drugs jasplakinolide and mycalolide B reduces invasion of both *T. gondii* and *P. berghei* parasites, indicating that host actin dynamics are important for parasite entry [24]. It was proposed that invading parasites reorganize host actin filaments to anchor the MJ to the underlying actin cortical network, in order to ensure junction stability [24]. Consistently, in both *T. gondii* and *P. berghei* invasion, the host actin-related 2/3 complex (Arp2/3), an actin nucleator [25], is actively recruited to the moving junction and is necessary for invasion, since its inhibition reduces the invasion rates of both parasites [24]. In order to facilitate parasite entry, *T. gondii* also induces a local relaxation of the cortical actin cytoskeleton. This is achieved through the acting of a rhoptry protein called toxofilin that directly binds actin filaments and regulates their turnover, thus loosening the filament network [26].

Moreover, *C. parvum* manipulates the host actin cytoskeleton during invasion, to induce the formation of a dense F-actin plaque at the site of parasite entry, by activating host calpains, proteases that remodel the actin cytoskeleton [27].

Most apicomplexans complete their whole intracellular growth inside the PV. Moreover, this step of the parasite life cycle requires a manipulation of the host cytoskeleton. For instance, *T. gondii* alters host microtubules during PV formation. Microtubules surround *T. gondii* PV soon after invasion and then the centrosome, the major microtubule organizing center, is detached from the host cell nuclear envelope and associated with the PV. Host microtubules could serve to provide mechanical support to the dividing parasites. Moreover, *T. gondii* shows microtubule-induced PV membrane (PVM) invaginations in the PV lumen that are used as conduits for the import of host nutrients and vesicles [18].

During their intracellular development, some apicomplexan parasites recruit the host organelles close to the PVM to scavenge host molecules or manipulate organelle activity to meet their own needs. They do so either by acting on the microtubule network that regulates organelle trafficking [21] or by creating a direct link with the organelles themselves. For instance, some *T. gondii* strains relocate the host mitochondria close to the PVM, probably to hijack their activity, even though it is still not well understood which benefits the parasite gains from doing so. It was shown that host microtubule disruption impairs the recruitment of host mitochondria [28]. *T. gondii* parasites secrete a protein called mitochondrial association factor 1 (MAF1) that binds to a host protein called mitochondrial intermembrane space bridging complex (MIB), causing an increase of the contact area between the PVM and the mitochondria that probably favors the import of metabolites to the PV [29,30].

Moreover, in order to scavenge lipids from the host, several apicomplexan parasites alter the host Golgi vesicle trafficking [31]. As an example, *T. gondii* localizes near to the host Golgi soon after invasion and then associates with it, causing its fragmentation in ministacks, thus increasing the contact area between the Golgi and the PV. The Golgi fragmentation and the proximity to the PV favor the interception of Golgi-derived vesicles that are internalized inside the PV through invaginations and evaginations of the PVM [14]. After crossing the PVM, they become encircled by membranous tubules forming an intravacuolar network inside the PV, where they are disassembled and release their content [15].

In addition, also *P. falciparum* manipulates the host intracellular trafficking, in this case to deliver its own proteins to the host cell surface. Malaria parasites export on the red blood cell surface sticky proteins in order to adhere to the microvascular endothelium and avoid the host splenic clearance. To do this, *P. falciparum* parasites produce in the host cytoplasm sack-like structures called “*Maurer’s clefts*” that are linked to the host actin cytoskeleton through the parasite trafficking protein 1 (PfPTP1). This protein stabilizes and elongates actin filaments that radiate from the parasite towards the host cell membrane, thus allowing the translocation of parasite proteins to the erythrocyte surface [32].

Once intracellular growth and replication have taken place, apicomplexan parasites need to egress from the host cell. Moreover, in this final step, host cytoskeleton modulation plays an important role. As an example, during *T. gondii* egress from the host cell, the parasite activates a host G-protein signaling pathway to dismantle the host cytoskeleton and facilitate egress. It was shown that *T. gondii* parasites activate the host Gq protein alpha subunit, which in turn activates protein kinase C (PKC). This kinase phosphorylates adducin, a protein that maintains erythrocyte cytoskeletal integrity, inhibiting its actin binding activity. Protein Gαq activation thus leads to adducin dissociation from the cytoskeleton, with a consequent loss of stability of the cytoskeleton itself, and the opening of the transient receptor potential cation channel 6 (TRPC6) on the plasma membrane, with a consequent influx of Ca^2+^ ions. Calcium activates host calpains, proteases that cleave several cytoskeletal substrates, leading to cytoskeleton dismantling and parasite egress. A similar strategy was also suggested for *P. falciparum* egress from the host cell [33].

Apicomplexan parasites thus frequently modify the host cytoskeleton by hijacking host signaling pathways involved in its regulation. In this review, we will focus on this specific aspect of host manipulation and in particular on the small GTPases superfamily, which includes some of the most studied examples of proteins regulating cytoskeleton organization and intracellular trafficking.

## 3. Roles of the Host Small GTPases in Intracellular Pathogen Infection

Small GTPases are a superfamily of small proteins of 21–30 kDa involved in signal transduction and characterized by a common “G domain”, which binds the nucleotide guanosine-triphosphate (GTP) and hydrolyzes it to guanosine-diphosphate (GDP). GTPases act like a switch, alternating between an active GTP-bound conformation, in which they bind downstream effectors, and a GDP-bound conformation, in which they are inactive. In the latter conformation, GDP will tend to dissociate from the GTPase, thus allowing a new GTP molecule to bind [34]. Interchange between GTP and GDP is regulated by different classes of GTPase interactors. Guanine nucleotide exchange factors (GEFs) promote GDP release and GTP loading, thus activating the signal. Each GTPase can interact with different GEFs, thus integrating signals from various sources. GTPase activating proteins (GAPs) bind to GTPases in their active conformation, improving their catalytic activity and allowing the protein to quickly return to its inactive state, thus shortening signal duration. Guanine-nucleotide-dissociation inhibitors (GDIs) maintain the GTPase in an off-state, preventing GDP dissociation, thus inhibiting the GTPase activity [35,36].

Small GTPases are divided into five families based on their sequence and structure: Rho, Rab, Ras, Arf and Ran families [37]. These proteins are exploited by all classes of intracellular pathogens both to invade the host cell and to develop and grow once inside the host. Bacteria have evolved different strategies to modulate the host small GTPases. The first consists of producing effectors that mimic GTPase modulators, such as GEFs, GAPs and GDIs. The best documented example of this strategy is the bacterium *Salmonella enterica*, a pathogen that has evolved a unique ability to up- and downregulate the activity of the host small GTPases Cdc42 and Rac1, two actin cytoskeleton key regulators. Upon invasion, *Salmonella* injects, through a needle-like appendage, two bacterially encoded GEFs, SopE and SopE2, inside the host cell. These proteins activate Cdc42 and Rac1, initiating a signal transduction cascade in the host cell that induces actin cytoskeletal rearrangements and membrane ruffling, aimed at facilitating bacterial engulfment and internalization [38]. The host cell morphological changes associated with bacteria uptake are rapidly reversed by *Salmonella* to ensure a minimally disrupted environment to live in. Once inside the host cell, these bacteria produce the secreted effector protein (SptP), a protein that antagonizes Cdc42 and Rac1 by mimicking a GAP and facilitates host cell actin cytoskeleton recovery [39]. SptP is the smallest GAP known so far and shows significant differences in its structure if compared to eukaryotic GAPs. Nevertheless, by analyzing the crystal structure of a SptP–Rac1 complex, it was shown that these differences are not exposed to the protein surface and SptP revealed an unusual GAP architecture that extensively mimics host functional homologs [40]. SptP constitutes an important example of convergent evolution between pathogen and host. Similar strategies have also been reported for many other bacteria that express factors mimicking all classes of GTPase modulators [41].

Another way widely used by intracellular pathogens to hijack the host small GTPases is by modifying them with post-translational modifications, such as ADP-ribosylation, glucosylation, deamidation and transglutamination, resulting either in their inactivation or activation. For instance, the bacterium *Clostridium botulinum* delivers into the host cell an exoenzyme called C3 that ADP-ribosylates the host small GTPase RhoA, inactivating its signaling pathway [42].

Another example of bacterial toxins modulating host small GTPases through post-translational modifications is the toxin cytotoxic necrotizing factor (CNF) from *Escherichia coli* and *Yersinia pseudotuberculosis*. CNF deaminates the host GTPases Rac1, RhoA and Cdc42, keeping them in an active state, thus stimulating actin polymerization and favoring bacterial entry [43].

Post-translational modifications can also modulate degradation of the host small GTPases through the ubiquitin–proteasome system. This is the case of a toxin called protein adenylyltransferase (VopS), expressed by *Vibrio parahaemolyticus*, that AMPylates Rac1 and RhoA, hindering their interaction with the E3 ubiquitin ligase, thus reducing their degradation. The consequent activation of Rac1 and RhoA signaling pathways inhibits several factors involved in the host response to bacterial infection [44]. On the contrary, the bacterium *Legionella pneumophila* secretes enzymes that ubiquitinate the host small GTPases Rab33b and Rab1, increasing their degradation in order to manipulate host vesicle trafficking to form a protective vacuole in which the bacterium will spend its intracellular life [45].

All classes of small GTPases were also shown to be involved in infection by viruses, at different stages of the viral life cycle, from cell entry [46,47,48,49,50,51], to replication [52,53,54], to late assembly/release of enveloped viruses [51].

Small GTPases are also involved in infection by some intracellular parasites. The three GTPases Rac1, Cdc42 and Arf6 were shown to be involved in host cell invasion by the flagellate parasite *Trypanosoma cruzi* [55,56], the etiological agent of Chagas disease. During *T. cruzi* invasion, these GTPases modulate the actin cytoskeleton at the site of parasite entry, favoring parasite penetration [55,56,57]. A similar mechanism was found also during invasion by the intracellular parasite *Leishmania donovani* that infects macrophages. Rac1 and Arf6 are activated during *L. donovani* entrance and mediate its phagocytosis by acting on actin modulation and on membrane recycling at the site of entry [58]. After invasion, Rac1 localizes to the phagosome where *L. donovani* resides and here, together with Cdc42, participates in the formation of an F-actin shell around the phagosome that arrests phagosomal maturation [59].

In the following sections, we will provide an overview on how apicomplexan parasites exploit the host small GTPases to their advantage.

## 4. Host Small GTPases in Apicomplexan Infection: The Roles of Rho GTPases

The Rho family comprises about 25 different members, involved in several functions, such as cytoskeleton modulation, cell polarity and cell cycle progression. They act as molecular switches in several pathways that link plasma membrane receptors to cytoskeletal reorganization. Many Rho GTPases can undergo prenylation on their C-terminal domain. This post-translational modification increases the protein hydrophobicity and promotes its subcellular localization to the plasma membrane [60]. Among Rho GTPases, the most studied are RhoA, Rac1 and Cdc42, due to their involvement in tumor progression and metastasis [61].

Because of their role in actin cytoskeleton modulation, GTPases belonging to the Rho family are involved in infection by many intracellular pathogens [40,48,49,50,58,59,62,63,64,65,66,67,68,69,70,71], including several apicomplexan parasites, such as *Plasmodia* [72,73,74,75], *T. gondii* [76,77], *C. parvum* [78,79] and *Theileria annulata* [80]. Moreover, several commercially available inhibitors of Rho GTPases have shown in vitro efficacy against intracellular pathogens, including bacteria [81,82], viruses [83,84] and protozoan parasites [85,86].

**Figure 1:**
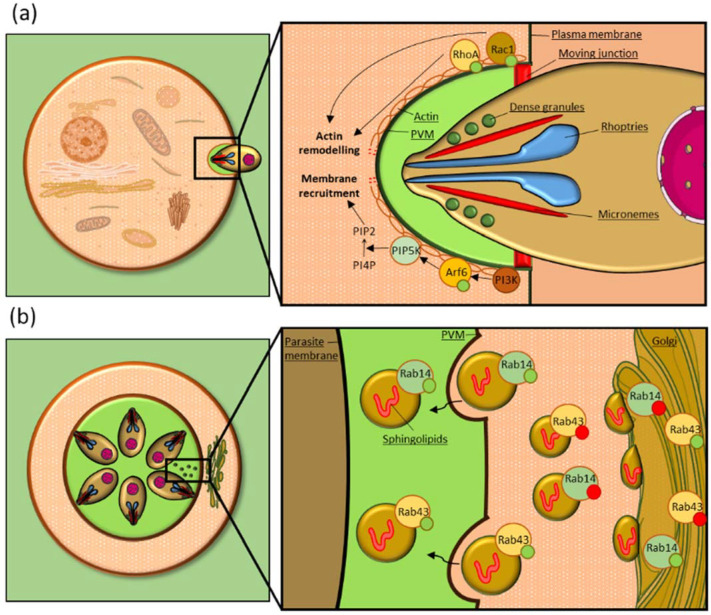
***Toxoplasma gondii* manipulates Rho, Arf and Rab GTPases**. (**a**) Schematic of an invading stage of *T. gondii*. Rac1 and RhoA are recruited to the site of parasite entry and their activity is required for actin cytoskeleton remodeling during invasion. Moreover, Arf6 is recruited to the site of parasite entry and activated by PI3-kinase. Arf6 activation triggers PIP5-kinase that produces PIP2 from PI4P, resulting in actin remodeling and membrane recruitment. (**b**) Schematic representing replicating *T. gondii* parasites. During their intracellular development, *T. gondii* parasites scavenge sphingolipids from host Golgi-derived vesicles bound to Rab14 and Rab43. Only vesicles bound to active Rab GTPases are delivered to the PV. PVM: parasitophorous vacuole membrane. Green circles represent GTP and red circles GDP. Figure created with the support of https://smart.servier.com/ (accessed on the 5 May 2022).

The Rho GTPase Rac1 was shown to play an important role in infection by both *T. gondii* and *P. falciparum*. *T. gondii* recruits Rac1 to its PVM and transfection of a Rac1 dominant-negative mutant (Rac1-N17) impairs the GTPase recruitment, indicating that its activation is required for mobilization to the PVM. The amount of active Rac1 was higher in infected cells compared to the uninfected control, suggesting that the parasite induces Rac1 activation. Moreover, both transfection of a Rac1 dominant-negative form and down-regulation of the endogenous protein by RNA interference led to a significant reduction in parasite invasion rates [76], showing that the GTPase is required for efficient invasion (Table 1, Figure 1a).

Recently, Wei et al. showed that host cell treatment with NSC23766 [93], a Rac1 specific inhibitor, reduces *T. gondii* invasion rates. Interestingly, treatment with this compound also reduces F-actin polymerization, suggesting that Rac1 activation may entail F-actin cytoskeleton modulation (Table 1, Figure 1a), as previously reported in infection by many other intracellular pathogens [46,47,48,55,62,64,66,78,82,94]. Finally, treatment of infected cells with NSC23766 during *T. gondii* intracellular development also reduced parasite replication rate, resulting in a significantly smaller parasite number compared to untreated cells. This suggests a possible role of the GTPase also during this phase of the parasite life cycle [77] (Table 1).

Rac1 plays a similar role also in *P. falciparum* infection of human erythrocytes (Table 1). We demonstrated that the GTPase is recruited to the PVM in infected erythrocytes and is activated by the parasite, similarly to *T. gondii*. We also showed that two Rac1 chemical inhibitors, EHT-1864 and 1A116 [95,96], reduced *P. falciparum* invasion rates, indicating an involvement of Rac1 in this process. Moreover, both inhibitors efficiently reduced parasitemia and parasite cell size, indicating an involvement of Rac1 also during *P. falciparum* intraerythrocytic development [72] (Table 1). When the parasite is mature, the GTPase is nearly totally depleted from the erythrocyte membrane [72,74]. It was suggested that Rac1 removal may be necessary for *P. falciparum* egress from the host cell. Loss of Rac1 is associated with adducin removal from the cytoskeleton and its subsequent destabilization [97]. Thus, the loss of Rac1 before parasite egress may help in dismantling the host cytoskeleton in preparation for subsequent disruption of the plasma membrane [74] (Table 1).

Because of its role in many types of cancer, Rac1 has been widely studied and several chemical inhibitors of the GTPase are commercially available and were shown to reduce invasion rates of many intracellular pathogens [49,64,66,68,98]. We tested several Rac1 inhibitory compounds, targeting either interaction with GEFs or nucleotide exchange activity, on *P. falciparum* in vitro cultures and showed that twelve inhibitors have antimalarial activity. Among them, three showed a half inhibitory concentration below 1 μM. The most efficient inhibitors were also tested for their cytotoxicity, giving good results on human microvascular endothelial cells (HMEC-1) [73].

The small GTPase Cdc42 was shown instead to be involved in *C. parvum* infection. *C. parvum* primarily infects intestinal epithelia but can also infect other types of epithelia [99]. When ingested, *C. parvum* oocysts excyst in the gastrointestinal tract and release infective sporozoites. The parasites then attach to the apical membrane of the host epithelial cells, inducing the formation of membrane protrusions that encapsulate the sporozoite and form an intracellular but extracytoplasmic PV [100].

It was shown that Cdc42 is recruited to the site of invasion, where it is activated. In order to activate Cdc42, *C. parvum* recruits to the site of parasite entry and activates phosphoinositide 3-kinase (PI3K), a signaling kinase implicated in actin polymerization. PI3K recruitment causes the accumulation of frabin, a GEF that activates Cdc42, finally leading to actin remodeling [79]. Both Cdc42 inhibition by overexpression of a dominant-negative mutant, and its suppression by small interfering RNA (siRNA) were associated with a reduction of *C. parvum*-associated actin remodeling and membrane protrusions and, ultimately, *C. parvum* invasion. Expression of a constitutively active Cdc42 mutant instead significantly increased *C. parvum* invasion. These findings demonstrated that Cdc42 plays an important role in *C. parvum* invasion of the host cell [78] (Table 1).

Cdc42 is also involved in infection by *T. annulata*, a tick-borne apicomplexan infecting ruminants. This parasite promotes uncontrolled proliferation and enhanced motility of its host macrophages through chronic de-regulation of their signaling pathways, thus promoting parasite dissemination in the host animal. Infected cells have often been used as a reversible model of oncogenic transformation since the parasite can be eliminated by parasiticide treatment [101]. The presence of intracellular *T. annulata* causes asymmetric activation of host cell actin dynamics and podosomes and lamellipodia formation. The asymmetrical rigidification of the cortical cytoskeleton at the rear increases contractility and intracellular pressure at the leading edge, where podosomes and lamellipodia form following activation of Rho kinases, a class of kinases activated by Rho GTPases. It was shown that Cdc42 activity was higher in virulent macrophages obtained ex vivo from the peripheral blood of calves infected with *T. annulata*, compared to attenuated cell lines or drug-cured macrophages previously infected, indicating that Cdc42 activity is increased during infection and decreases upon parasite elimination [80]. It was also shown that tumor necrosis factor alpha (TNFα) is overexpressed in virulent macrophages [102]. Since in other cell types TNFα promotes filipodia formation via the Cdc42 pathway [103], it is possible that this GTPase could be responsible for filipodia formation also in *Theileria*-infected macrophages [80] (Table 1).

RhoA is another key regulator of actin dynamics that is manipulated by Apicomplexa. During *T. gondii* invasion of the host cell, RhoA is recruited to the site of parasite entry at the beginning of the invasion process and is subsequently relocated to the PVM in its active form. Moreover, both the transfection of a dominant-negative form of RhoA and its silencing by RNA interference reduced *T. gondii* invasion rates [76] (Table 1, Figure 1a).

In addition to their role in host cell infection, small GTPases were found to be involved in *P. falciparum*-infected erythrocytes’ adherence to the vascular endothelium, a key factor in the occurrence of severe malaria. It was shown that treatment of endothelial cells with *E. coli* toxin CNF1 [104], a Rho/Rac1/Cdc42 modulator, both prevents cytoadherence and induces the detachment of infected erythrocytes from endothelial cells in vitro by activation of RhoA, Cdc42 and Rac1. Therefore, CNF1 was proposed as a candidate for the development of treatments against severe malaria [75] (Table 1).

## 5. Host Small GTPases in Apicomplexan Infections: The Roles of Rab GTPases

The Rab family is the largest group of small GTPases, with more than 60 members localized to different intracellular membranes. They are characterized by a reversible association with membranes through geranylgeranyl groups that are attached to their C-terminal end. Rab GTPases are involved in the modulation of vesicular trafficking and protein transport. Each intracellular membrane is characterized by specific Rab proteins [37,105].

Apicomplexan parasites manipulate Rab GTPases by promoting or antagonizing their function, in order to modulate cellular trafficking pathways, move host vesicles near to or inside their PVM, scavenge nutrients from host vesicles and control host phagocytic activity [31].

For instance, *T. gondii* preferentially internalizes vesicles involved in Golgi trafficking, in order to scavenge sphingolipids and other nutrients from the host, which the parasite is auxotrophic for [15]. To facilitate this, some *T. gondii* strains relocate the host Golgi near the PV and fragment it in ministacks [14].

In particular these parasites are able to selectively recognize and internalize to their PV vesicles bound to specific Rab GTPases [15]. Among them, Rab14 and Rab43, two Golgi GTPases, are a clear example of Rab manipulation for parasite needs. When dominant negative Rab14 and Rab43 mutants were expressed in the host cells, sphingolipids showed an aberrant localization in large foci on and within the PVM. Moreover, the sphingolipid amount in the PV lumen was significantly reduced compared to the control, indicating that Rab14 and Rab43 activity is required for an efficient uptake of host sphingolipids [14]. It was also shown that the GTP-bound form of Rab14 is preferentially internalized to the PV, compared to the GDP-bound form, confirming that GTPase activation is required for vesicle delivery to the PV lumen [15] (Table 1, Figure 1b). This mechanism has already been shown for other Rabs, such as Rab1 (Table 1), suggesting this may be a common feature in Rab vesicle internalization [15].

Moreover, *P. berghei* exploits host Rab GTPases to acquire nutrients from the host hepatocyte. Like *T. gondii*, *P. berghei* fragments the host cell Golgi, in order to increase the surface interaction between this organelle and the PVM. It was shown that transfection of a dominant-negative mutant of Rab11a, a GTPase that regulates endosome recycling in the trans-Golgi, prevents the Golgi fragmentation exerted by the parasite and significantly reduces both the number of parasites reaching maturation and parasite size [12] (Table 1).

Rab GTPases are also key regulators of vesicular trafficking in the endocytic pathways in macrophages [106]. Some apicomplexan parasites can interfere with these proteins in order to subvert the host immune response [31].

For instance, *P. berghei* parasites modulate Rab gene transcription in macrophages to their own advantage. It was shown that when macrophages were incubated with *P. berghei*-infected erythrocytes, the expression levels of nine different Rabs regulating phagocytosis increased. In particular, Rab14 plays a protective role for the parasite, since its silencing causes a two-fold increase in phagocytosed parasites, while its overexpression causes a significant decrease [88] (Table 1). In macrophages, Rab14 is involved in the trafficking to the plasma membrane of CD36, a receptor involved in *P. berghei* phagocytosis. As a matter of fact, it was shown that its silencing leads to an increase in CD36 surface expression due to a reduction in its internalization [89]. It was proposed that the primary uptake of *P. berghei*-infected erythrocytes increases the levels of Rab14, leading to a reduction in CD36 receptor exposed on the macrophage surface, thus inhibiting subsequent phagocytic activity and decreasing parasite clearance [88,89].

## 6. Host Small GTPases in Apicomplexan Infection: The Roles of Ras, Arf and Ran GTPases

The Ras family includes GTPases with a high degree of sequence identity and function redundancy that are important regulators of signaling pathways linking extracellular signals to gene transcription, cell proliferation, differentiation and morphology. The Ras genes were the first small GTPase genes to be identified and are the most common oncogenes in human cancer [107].

*T. gondii* parasites infect a variety of human cells, including dendritic cells (DCs), antigen-presenting cells that trigger adaptive immune response. Infected DCs show a hypermigratory phenotype characterized by amoeboid motility that guarantees parasite dissemination inside the host organism. In order to promote hypermotility, *T. gondii* induces gamma-aminobutyric acid (GABA) secretion and activation of its ionotropic receptor, leading to calcium influx. The increase in Ca^2+^ concentration induces the activation of calmodulin (CaM) and CaM kinase II (CaMkII), which in turn activates the GTPase Ras. Moreover, the MET receptor tyrosine kinase is activated upon *T. gondii* infection and its signaling also activates Ras, which is thus a nodal factor coordinating signals from both GABA and MET receptors. Ras activation leads to extracellular signal-regulated kinase 1/2 (Erk1/2) phosphorylation, which in turn phosphorylates proteins in the nucleus and in the cytoplasm that maintain hypermotility [90] (Table 1).

The ADP-ribosylation factor family (Arf) is composed of six proteins with overlapping functions, characterized by an N-terminal amphipathic domain of 14 amino acids that is myristoylated. When the Arf GTPase is bound to GDP, the N-terminal domain faces towards the catalytic site and is autoinhibitory. When the protein is bound by a GEF, the myristoylated domain swings out of the protein and ensures its association to membranes, allowing GTP binding and consequent GTPase activation.

Arf GTPases mainly regulate organelle structure, membrane biosynthesis, trafficking and interaction with the actin cytoskeleton, lipid transport in the Golgi network and recruitment of proteins that regulate cargo sorting into vesicles. Arfs localize to membranes throughout the cell, including the plasma membrane and the membranes of the secretory, endosomal and lysosomal pathways [108,109].

Arf6, a GTPase involved in membrane trafficking during endocytosis [110], was shown to be involved in *T. gondii* invasion of the host cell. The GTPase is recruited to the PV of invading parasites, and when its activity is inhibited by transfecting the host cell with a dominant-negative form of the GTPase, or when the endogenous Arf6 is inactivated by RNA interference, parasite invasion rates decrease. This indicates that Arf6 is important for *T. gondii* invasion of the host cell, possibly playing a role in membrane invagination and PV formation. In this process, Arf6 is triggered by phosphatidylinositol 3-kinase (PI3-kinase), a kinase that is activated upon invasion. In turn, Arf6 activates phosphatidylinositol 4-phosphate 5-kinase (PIP5-kinase) that is responsible for generating phosphatidylinositol 4,5-biphosphate (PIP2), which surrounds the invading parasite and is known to regulate actin cytoskeleton dynamics [91] (Table 1, Figure 1a).

The Ran family includes only one member, the protein Ran, characterized by an acidic tail at its C-terminus and by the absence of a membrane-binding motif, present in all the other small GTPases. Ran, unlike the other GTPases that are either cytoplasmic or associated with membranes, localizes both in the nucleus and the cytoplasm. Ran is involved in the regulation of several cellular functions, such as cell cycle progression through the regulation of the cell spindle apparatus and nuclear envelope formation during mitosis, cytoplasm–nucleoplasm transport and RNA nuclear export, and synthesis and processing during interphase [37,111].

The only apicomplexan parasite that was shown to manipulate Ran is *T. annulata*. This is not surprising, since *Theileria* parasites deeply alter the host cell cycle, differentiation and motility to favor their spread in the host organism [101]. They do so both by modulating the host gene transcription and by manipulating its signaling pathways.

These parasites, unlike other apicomplexan, are free in the host cytoplasm and are not surrounded by a PV. In infected cells, the parasite forms the so-called annulate lamellae (AL), a membranous structure localized in close proximity to the parasite, containing pores similar to the nuclear pore complexes (NPCs) [112]. It was recently demonstrated that the components of the AL, including Ran, are in fact derived from the host NPCs. The role of Ran may be linked to the delivery of soluble parasite factors to the host nucleus, in order to subvert gene transcription [92]. Interestingly, two proteins that regulate Ran, the Ran GTPase-activating protein 1 (RanGAP1) and the Ran-binding protein 2 (RanBP2), which are both part of the NPCs, were found to associate with *Theileria* parasites, thus indicating some sort of regulation of host Ran GTPase in proximity to the parasite surface [113] (Table 1).

## 7. Discussion and Conclusions

Small GTPases are key regulators of virtually all aspects of eukaryotic cell life. Given the extensive interplay between apicomplexan parasites and host functions, these proteins are manipulated during all stages of apicomplexan infections, from invasion of the host cell to intracellular growth and egress.

Exploitation of host small GTPases by apicomplexan parasites is a clear example of adaptation to intracellular parasitism based on the manipulation of host cell functions in order to favor the pathogen’s survival and spreading.

The emergence of drug-resistant strains in several species of Apicomplexa is a major threat in the fight against these pathogens. Drug-resistant strains of *P. falciparum* have by now been reported against all classes of antimalarial compounds [114,115]. Resistant strains of *T. gondii* are emerging worldwide, representing a risk of increased disease severity or treatment failure in immunocompromised patients [116]. Regarding *C. parvum*, vaccines or fully effective drugs are not yet available and anti-cryptosporidial drug discovery is underway. The only drug approved by the Food and Drug Administration, nitazoxanide, is efficient only to treat cryptosporidiosis in immuno-competent individuals, but not in immuno-compromised ones, which are at risk of severe disease [117].

An emerging strategy to limit the insurgence of drug resistance is to target host molecules exploited by pathogens to enter or develop inside the host cell. This approach is less likely to generate drug resistance compared to pathogen-directed therapies, since the pathogens would need to modify their entire infection strategy to compensate for a missing host factor. Moreover, host-targeted therapies (HTTs) could be effective against different parasite strains [118,119]. Given the high dependence of apicomplexan parasites on host cell functions, HTTs are a promising strategy for the treatment of apicomplexan infections.

Host-targeted molecules have already been proven effective against several intracellular pathogens [120,121,122,123]; some have been tested in clinical trials, for instance against *Hepatitis C* [124], and some are already in clinical use for *HIV* treatment [125]. In apicomplexan infections, this strategy has been proposed for malaria and toxoplasmosis [126,127]. Treatment of human erythrocytes with Conoidin A, an inhibitor of the host peroxidase Prx2, impairs parasite DNA synthesis and hemozoin production, disrupts nuclear integrity and prevents *P. falciparum* growth [128]. Moreover, human ferrochelatase, the final enzyme of the heme biosynthetic pathway, has been proposed as a suitable target for the development of novel HTTs, since *P. falciparum* growth is impaired in erythrocytes treated with N-methylprotoporphyrin (N-MPP), a ferrochelatase substrate analogue and competitive inhibitor of the enzyme. Moreover, the growth of *Plasmodium* intraerythrocytic stages is inhibited both in human red blood cells from individuals with natural loss of function mutations of the protein and in mice carrying a ferrochelatase knockdown mutation. The fact that individuals with natural loss of function mutations of ferrochelatase display relatively few symptoms and have a normal life expectancy suggests that targeting ferrochelatase would be well tolerated by the organism [129]. Imiquimod is an immune response modulating drug that was shown to be efficient both as a therapy and as a prophylaxis against *T. gondii* both in vitro and in vivo in murine models, acting both during acute and chronic toxoplasmosis. This compound binds the host Toll-like receptor-7 (TLR-7), activating its downstream signaling pathway, thus leading to innate immune response activation [130].

In our recent work, we proposed the Rho GTPase Rac1 as a potential host target for the development of novel antimalarial drugs [72,73] and EHop-016, the most efficient Rac1 inhibitor against *P. falciparum*, as a promising starting molecule for the development of novel, more effective compounds [131,132]. Moreover, the Rho modulator CNF from *E. coli* was proposed as a treatment against severe malaria, specifically targeting the adhesion of infected erythrocytes to endothelia [75].

The availability of several small GTPase inhibitors [133,134] allows for quick and easy testing of their efficacy against clinically relevant apicomplexan parasites in the search for novel host-targeted compounds. Screening of inhibitory compounds in vitro against apicomplexan parasites is easily conceivable, given the availability of robust protocols for drug susceptibility assessment for all the clinically relevant apicomplexan parasites [135,136,137].

Moreover, the repurposing of drugs in use for other human diseases is a promising strategy, with the benefits of being more cost-effective and requiring less time compared to the development of novel drugs, since data on pharmacokinetics and toxicity are already available [138,139]. For instance, the immunosuppressive drug azathioprine, used in the treatment of some autoimmune diseases, as well as the potent analgesic R-ketorolac, were shown to be Rho GTPase inhibitors [140,141]. Moreover, azathioprine was also shown to exert antimalarial activity both in vitro on *P. falciparum* cultures and in vivo on *P. berghei*-infected mice [142].

Given the wide distribution of small GTPases in different human tissues and their involvement in many cellular functions, therapies based on their inhibition could potentially cause collateral effects [133]. A deeper understanding of the mechanisms by which pathogens exploit the host small GTPases could obviate this risk, by allowing the design of compounds targeting specific host–parasite interactions, which would be more effective and less toxic and could even lead to the identification of novel therapeutic targets.

## Figures and Tables

**Table 1:** Summary table of the host small GTPases exploited by apicomplexan parasites.

Small GTPases Family	GTPase	Function	Organism
Rho	Rac1	Invasion	*P. falciparum* [72,73]
		Intracellular Growth	*P. falciparum* [72,73], *T. gondii* [77]
		Egress	*P. falciparum* [74]
		Cytoadherence	*P. falciparum* [75]
	Cdc42	Invasion	*C. parvum* [78,79]
		Cytoadherence	*P. falciparum* [75]
		Infected cell motility	*T. annulate* [80]
	RhoA	Invasion	*T. gondii* [76]
		Cytoadherence	*P. falciparum* [75,87]
Rab	Rab1	Nutrients scavenging	*T. gondii* [15]
	Rab11	Nutrients scavenging	*P. berghei* [12]
	Rab14	Nutrients scavenging	*T. gondii* [14]
		Immune evasion	*P. berghei* [88,89]
	Rab43	Nutrients scavenging	*T. gondii* [14]
Ras	Ras	Infected cell motility	*T. gondii* [90]
Arf	Arf6	Infected cell motility	*T. gondii* [91]
Ran	Ran	Nuclear delivery of parasite factors	*T. annulate* [92]

## Data Availability

Not applicable.

## References

[1] WHO (2022). World Malaria Report 2021.

[2] Bigna J.J., Tochie J.N., Tounouga D.N., Bekolo A.O., Ymele N.S., Youda E.L., Sime P.S., Nansseu J.R. (2020). Global, regional, and country seroprevalence of *Toxoplasma gondii* in pregnant women: A systematic review, modelling and meta-analysis. Sci. Rep..

[3] Basavaraju A. (2016). Toxoplasmosis in HIV infection: An overview. Trop. Parasitol..

[4] Gerace E., Lo Presti V.D.M., Biondo C. (2019). Infection: Epidemiology, Pathogenesis, and Differential Diagnosis. Eur. J. Microbiol. Immunol..

[5] Gubbels M.J., Duraisingh M.T. (2012). Evolution of apicomplexan secretory organelles. Int. J. Parasitol..

[6] Adl S.M., Leander B.S., Simpson A.G., Archibald J.M., Anderson O.R., Bass D., Bowser S.S., Brugerolle G., Farmer M.A., Karpov S. (2007). Diversity, nomenclature, and taxonomy of protists. Syst. Biol..

[7] Krishnan A., Soldati-Favre D. (2021). Amino Acid Metabolism in Apicomplexan Parasites. Metabolites.

[8] Shunmugam S., Arnold C.S., Dass S., Katris N.J., Botté C.Y. (2022). The flexibility of Apicomplexa parasites in lipid metabolism. PLoS Pathog..

[9] Counihan N.A., Modak J.K., de Koning-Ward T.F. (2021). How Malaria Parasites Acquire Nutrients From Their Host. Front. Cell Dev. Biol..

[10] Cardoso R., Nolasco S., Gonçalves J., Cortes H.C., Leitão A., Soares H. (2014). Besnoitia besnoiti and *Toxoplasma gondii*: Two apicomplexan strategies to manipulate the host cell centrosome and Golgi apparatus. Parasitology.

[11] Cardoso R., Wang J., Müller J., Rupp S., Leitão A., Hemphill A. (2018). Modulation of cis- and trans- Golgi and the Rab9A-GTPase during infection by Besnoitia besnoiti, *Toxoplasma gondii* and Neospora caninum. Exp. Parasitol..

[12] De Niz M., Caldelari R., Kaiser G., Zuber B., Heo W.D., Heussler V.T., Agop-Nersesian C. (2021). Hijacking of the host cell Golgi by Plasmodium berghei liver stage parasites. J. Cell Sci..

[13] Kellermann M., Scharte F., Hensel M. (2021). Manipulation of Host Cell Organelles by Intracellular Pathogens. Int. J. Mol. Sci..

[14] Romano J.D., Sonda S., Bergbower E., Smith M.E., Coppens I. (2013). Toxoplasma gondii salvages sphingolipids from the host Golgi through the rerouting of selected Rab vesicles to the parasitophorous vacuole. Mol. Biol. Cell.

[15] Romano J.D., Nolan S.J., Porter C., Ehrenman K., Hartman E.J., Hsia R.C., Coppens I. (2017). The parasite Toxoplasma sequesters diverse Rab host vesicles within an intravacuolar network. J. Cell Biol..

[16] Wang Y., Weiss L.M., Orlofsky A. (2010). Coordinate control of host centrosome position, organelle distribution, and migratory response by *Toxoplasma gondii* via host mTORC2. J. Biol. Chem..

[17] Coppens I., Dunn J.D., Romano J.D., Pypaert M., Zhang H., Boothroyd J.C., Joiner K.A. (2006). Toxoplasma gondii sequesters lysosomes from mammalian hosts in the vacuolar space. Cell.

[18] Cardoso R., Soares H., Hemphill A., Leitão A. (2016). Apicomplexans pulling the strings: Manipulation of the host cell cytoskeleton dynamics. Parasitology.

[19] Plattner F., Soldati-Favre D. (2008). Hijacking of host cellular functions by the Apicomplexa. Annu. Rev. Microbiol..

[20] Colonne P.M., Winchell C.G., Voth D.E. (2016). Hijacking Host Cell Highways: Manipulation of the Host Actin Cytoskeleton by Obligate Intracellular Bacterial Pathogens. Front. Cell. Infect. Microbiol..

[21] Frénal K., Soldati-Favre D. (2009). Role of the parasite and host cytoskeleton in apicomplexa parasitism. Cell Host. Microbe.

[22] Sweeney K.R., Morrissette N.S., LaChapelle S., Blader I.J. (2010). Host cell invasion by *Toxoplasma gondii* is temporally regulated by the host microtubule cytoskeleton. Eukaryot. Cell.

[23] Takemae H., Sugi T., Kobayashi K., Gong H., Ishiwa A., Recuenco F.C., Murakoshi F., Iwanaga T., Inomata A., Horimoto T. (2013). Characterization of the interaction between *Toxoplasma gondii* rhoptry neck protein 4 and host cellular β-tubulin. Sci. Rep..

[24] Gonzalez V., Combe A., David V., Malmquist N.A., Delorme V., Leroy C., Blazquez S., Ménard R., Tardieux I. (2009). Host cell entry by apicomplexa parasites requires actin polymerization in the host cell. Cell Host Microbe.

[25] Chhabra E.S., Higgs H.N. (2007). The many faces of actin: Matching assembly factors with cellular structures. Nat. Cell Biol..

[26] Delorme-Walker V., Abrivard M., Lagal V., Anderson K., Perazzi A., Gonzalez V., Page C., Chauvet J., Ochoa W., Volkmann N. (2012). Toxofilin upregulates the host cortical actin cytoskeleton dynamics, facilitating Toxoplasma invasion. J. Cell Sci..

[27] Perez-Cordon G., Nie W., Schmidt D., Tzipori S., Feng H. (2011). Involvement of host calpain in the invasion of *Cryptosporidium parvum*. Microbes Infect..

[28] Sinai A.P., Webster P., Joiner K.A. (1997). Association of host cell endoplasmic reticulum and mitochondria with the *Toxoplasma gondii* parasitophorous vacuole membrane: A high affinity interaction. J. Cell Sci..

[29] Pernas L., Adomako-Ankomah Y., Shastri A.J., Ewald S.E., Treeck M., Boyle J.P., Boothroyd J.C. (2014). Toxoplasma effector MAF1 mediates recruitment of host mitochondria and impacts the host response. PLoS Biol..

[30] Kelly F.D., Wei B.M., Cygan A.M., Parker M.L., Boulanger M.J., Boothroyd J.C. (2017). MAF1b Binds the Host Cell MIB Complex To Mediate Mitochondrial Association. mSphere.

[31] Coppens I., Romano J.D. (2020). Sitting in the driver’s seat: Manipulation of mammalian cell Rab GTPase functions by apicomplexan parasites. Biol. Cell.

[32] Rug M., Cyrklaff M., Mikkonen A., Lemgruber L., Kuelzer S., Sanchez C.P., Thompson J., Hanssen E., O’Neill M., Langer C. (2014). Export of virulence proteins by malaria-infected erythrocytes involves remodeling of host actin cytoskeleton. Blood.

[33] Millholland M.G., Mishra S., Dupont C.D., Love M.S., Patel B., Shilling D., Kazanietz M.G., Foskett J.K., Hunter C.A., Sinnis P. (2013). A host GPCR signaling network required for the cytolysis of infected cells facilitates release of apicomplexan parasites. Cell Host Microbe.

[34] Vetter I.R., Wittinghofer A. (2001). The guanine nucleotide-binding switch in three dimensions. Science.

[35] Buchsbaum R.J. (2007). Rho activation at a glance. J. Cell Sci..

[36] Scheffzek K., Ahmadian M.R. (2005). GTPase activating proteins: Structural and functional insights 18 years after discovery. Cell. Mol. Life Sci. CMLS.

[37] Song S., Cong W., Zhou S., Shi Y., Dai W., Zhang H., Wang X., He B., Zhang Q. (2019). Small GTPases: Structure, biological function and its interaction with nanoparticles. Asian J. Pharm. Sci..

[38] Hardt W.D., Chen L.M., Schuebel K.E., Bustelo X.R., Galán J.E. (1998). *S. typhimurium* encodes an activator of Rho GTPases that induces membrane ruffling and nuclear responses in host cells. Cell.

[39] Fu Y., Galán J.E. (1999). A salmonella protein antagonizes Rac-1 and Cdc42 to mediate host-cell recovery after bacterial invasion. Nature.

[40] Stebbins C.E., Galán J.E. (2000). Modulation of host signaling by a bacterial mimic: Structure of the Salmonella effector SptP bound to Rac1. Mol. Cell.

[41] Popoff M.R. (2014). Bacterial factors exploit eukaryotic Rho GTPase signaling cascades to promote invasion and proliferation within their host. Small GTPases.

[42] Barth H., Olenik C., Sehr P., Schmidt G., Aktories K., Meyer D.K. (1999). Neosynthesis and activation of Rho by *Escherichia coli* cytotoxic necrotizing factor (CNF1) reverse cytopathic effects of ADP-ribosylated Rho. J. Biol. Chem..

[43] Blumenthal B., Hoffmann C., Aktories K., Backert S., Schmidt G. (2007). The cytotoxic necrotizing factors from Yersinia pseudotuberculosis and from *Escherichia coli* bind to different cellular receptors but take the same route to the cytosol. Infect. Immun..

[44] Woolery A.R., Yu X., LaBaer J., Orth K. (2014). AMPylation of Rho GTPases subverts multiple host signaling processes. J. Biol. Chem..

[45] Qiu J., Sheedlo M.J., Yu K., Tan Y., Nakayasu E.S., Das C., Liu X., Luo Z.Q. (2016). Ubiquitination independent of E1 and E2 enzymes by bacterial effectors. Nature.

[46] Zamudio-Meza H., Castillo-Alvarez A., González-Bonilla C., Meza I. (2009). Cross-talk between Rac1 and Cdc42 GTPases regulates formation of filopodia required for dengue virus type-2 entry into HMEC-1 cells. J. Gen. Virol..

[47] Wang J.L., Zhang J.L., Chen W., Xu X.F., Gao N., Fan D.Y., An J. (2010). Roles of small GTPase Rac1 in the regulation of actin cytoskeleton during dengue virus infection. PLoS Negl. Trop. Dis..

[48] Pontow S.E., Heyden N.V., Wei S., Ratner L. (2004). Actin cytoskeletal reorganizations and coreceptor-mediated activation of rac during human immunodeficiency virus-induced cell fusion. J. Virol..

[49] Zoughlami Y., Voermans C., Brussen K., van Dort K.A., Kootstra N.A., Maussang D., Smit M.J., Hordijk P.L., van Hennik P.B. (2012). Regulation of CXCR4 conformation by the small GTPase Rac1: Implications for HIV infection. Blood.

[50] Swaine T., Dittmar M.T. (2015). CDC42 Use in Viral Cell Entry Processes by RNA Viruses. Viruses.

[51] Spearman P. (2018). Viral interactions with host cell Rab GTPases. Small GTPases.

[52] Belov G.A., Habbersett C., Franco D., Ehrenfeld E. (2007). Activation of cellular Arf GTPases by poliovirus protein 3CD correlates with virus replication. J. Virol..

[53] Zhang Q., Gong R., Qu J., Zhou Y., Liu W., Chen M., Liu Y., Zhu Y., Wu J. (2012). Activation of the Ras/Raf/MEK pathway facilitates hepatitis C virus replication via attenuation of the interferon-JAK-STAT pathway. J. Virol..

[54] Porter F.W., Bochkov Y.A., Albee A.J., Wiese C., Palmenberg A.C. (2006). A picornavirus protein interacts with Ran-GTPase and disrupts nucleocytoplasmic transport. Proc. Natl. Acad. Sci. USA.

[55] Bonfim-Melo A., Ferreira É., Mortara R.A. (2018). Rac1/WAVE2 and Cdc42/N-WASP Participation in Actin-Dependent Host Cell Invasion by Extracellular Amastigotes of. Front. Microbiol..

[56] Teixeira T.L., Cruz L., Mortara R.A., Da Silva C.V. (2015). Revealing Annexin A2 and ARF-6 enrollment during Trypanosoma cruzi extracellular amastigote-host cell interaction. Parasit. Vectors.

[57] Dutra J.M.F., Bonilha V.L., De Souza W., Carvalho T.M.U. (2005). Role of small GTPases in Trypanosoma cruzi invasion in MDCK cell lines. Parasitol. Res..

[58] Lodge R., Descoteaux A. (2006). Phagocytosis of *Leishmania donovani* amastigotes is Rac1 dependent and occurs in the absence of NADPH oxidase activation. Eur. J. Immunol..

[59] Lerm M., Holm A., Seiron A., Särndahl E., Magnusson K.E., Rasmusson B. (2006). *Leishmania donovani* requires functional Cdc42 and Rac1 to prevent phagosomal maturation. Infect. Immun..

[60] Mitin N., Roberts P.J., Chenette E.J., Der C.J. (2012). Posttranslational lipid modification of Rho family small GTPases. Methods Mol. Biol..

[61] Wennerberg K., Der C.J. (2004). Rho-family GTPases: It’s not only Rac and Rho (and I like it). J. Cell Sci..

[62] Billker O., Popp A., Brinkmann V., Wenig G., Schneider J., Caron E., Meyer T.F. (2002). Distinct mechanisms of internalization of Neisseria gonorrhoeae by members of the CEACAM receptor family involving Rac1- and Cdc42-dependent and -independent pathways. EMBO J..

[63] Boehm M., Krause-Gruszczynska M., Rohde M., Tegtmeyer N., Takahashi S., Oyarzabal O.A., Backert S. (2011). Major host factors involved in epithelial cell invasion of Campylobacter jejuni: Role of fibronectin, integrin beta1, FAK, Tiam-1, and DOCK180 in activating Rho GTPase Rac1. Front. Cell Infect. Microbiol..

[64] Ford C., Nans A., Boucrot E., Hayward R.D. (2018). Chlamydia exploits filopodial capture and a macropinocytosis-like pathway for host cell entry. PLoS Pathog..

[65] Friebel A., Ilchmann H., Aepfelbacher M., Ehrbar K., Machleidt W., Hardt W.D. (2001). SopE and SopE2 from Salmonella typhimurium activate different sets of RhoGTPases of the host cell. J. Biol. Chem..

[66] Kim H., White C.D., Li Z., Sacks D.B. (2011). Salmonella enterica serotype Typhimurium usurps the scaffold protein IQGAP1 to manipulate Rac1 and MAPK signalling. Biochem. J..

[67] Krause-Gruszczynska M., Rohde M., Hartig R., Genth H., Schmidt G., Keo T., König W., Miller W.G., Konkel M.E., Backert S. (2007). Role of the small Rho GTPases Rac1 and Cdc42 in host cell invasion of Campylobacter jejuni. Cell. Microbiol..

[68] Maruvada R., Zhu L., Pearce D., Zheng Y., Perfect J., Kwon-Chung K.J., Kim K.S. (2012). Cryptococcus neoformans phospholipase B1 activates host cell Rac1 for traversal across the blood-brain barrier. Cell. Microbiol..

[69] Prehna G., Ivanov M.I., Bliska J.B., Stebbins C.E. (2006). Yersinia virulence depends on mimicry of host Rho-family nucleotide dissociation inhibitors. Cell.

[70] Burnham C.A., Shokoples S.E., Tyrrell G.J. (2007). Rac1, RhoA, and Cdc42 participate in HeLa cell invasion by group B streptococcus. FEMS Microbiol. Lett..

[71] Doye A., Mettouchi A., Bossis G., Clément R., Buisson-Touati C., Flatau G., Gagnoux L., Piechaczyk M., Boquet P., Lemichez E. (2002). CNF1 exploits the ubiquitin-proteasome machinery to restrict Rho GTPase activation for bacterial host cell invasion. Cell.

[72] Paone S., D’Alessandro S., Parapini S., Celani F., Tirelli V., Pourshaban M., Olivieri A. (2020). Characterization of the erythrocyte GTPase Rac1 in relation to *Plasmodium falciparum* invasion. Sci. Rep..

[73] Parapini S., Paone S., Erba E., Cavicchini L., Pourshaban M., Celani F., Contini A., D’Alessandro S., Olivieri A. (2021). In vitro antimalarial activity of inhibitors of the human GTPase Rac1. Antimicrob. Agents Chemother..

[74] Millholland M.G., Chandramohanadas R., Pizzarro A., Wehr A., Shi H., Darling C., Lim C.T., Greenbaum D.C. (2011). The malaria parasite progressively dismantles the host erythrocyte cytoskeleton for efficient egress. Mol. Cell. Proteom..

[75] Messina V., Loizzo S., Travaglione S., Bertuccini L., Condello M., Superti F., Guidotti M., Alano P., Silvestrini F., Fiorentini C. (2019). The bacterial protein CNF1 as a new strategy against *Plasmodium falciparum* cytoadherence. PLoS ONE.

[76] Na R.-H., Zhu G.-H., Luo J.-X., Meng X.-J., Cui L., Peng H.-J., Chen X.-g., Gomez-Cambronero J. (2013). Enzymatically active Rho and Rac small-GTPases are involved in the establishment of the vacuolar membrane after *Toxoplasma gondii* invasion of host cells. BMC Microbiol..

[77] Wei H., Zhou L., Wu S., Li D., Deng S., Peng H. (2020). Host cell Rac1 GTPase facilitates *Toxoplasma gondii* invasion. Sci. China Life Sci..

[78] Chen X.M., Huang B.Q., Splinter P.L., Orth J.D., Billadeau D.D., McNiven M.A., LaRusso N.F. (2004). Cdc42 and the actin-related protein/neural Wiskott-Aldrich syndrome protein network mediate cellular invasion by *Cryptosporidium parvum*. Infect. Immun..

[79] Chen X.M., Splinter P.L., Tietz P.S., Huang B.Q., Billadeau D.D., LaRusso N.F. (2004). Phosphatidylinositol 3-kinase and frabin mediate *Cryptosporidium parvum* cellular invasion via activation of Cdc42. J. Biol. Chem..

[80] Ma M., Baumgartner M. (2013). Filopodia and membrane blebs drive efficient matrix invasion of macrophages transformed by the intracellular parasite *Theileria annulata*. PLoS ONE.

[81] Schmeck B., Beermann W., van Laak V., Opitz B., Hocke A.C., Meixenberger K., Eitel J., Chakraborty T., Schmidt G., Barth H. (2006). Listeria monocytogenes induced Rac1-dependent signal transduction in endothelial cells. Biochem. Pharmacol..

[82] Agarwal V., Hammerschmidt S. (2009). Cdc42 and the phosphatidylinositol 3-kinase-Akt pathway are essential for PspC-mediated internalization of pneumococci by respiratory epithelial cells. J. Biol. Chem..

[83] Zhang F., Liu Y., You Q., Yang E., Liu B., Wang H., Xu S., Nawaz W., Chen D., Wu Z. (2021). NSC23766 and Ehop016 Suppress Herpes Simplex Virus-1 Replication by Inhibiting Rac1 Activity. Biol. Pharm. Bull..

[84] Dierkes R., Warnking K., Liedmann S., Seyer R., Ludwig S., Ehrhardt C. (2014). The Rac1 inhibitor NSC23766 exerts anti-influenza virus properties by affecting the viral polymerase complex activity. PLoS ONE.

[85] Lodge R., Descoteaux A. (2005). *Leishmania donovani* promastigotes induce periphagosomal F-actin accumulation through retention of the GTPase Cdc42. Cell Microbiol..

[86] Morehead J., Coppens I., Andrews N.W. (2002). Opsonization modulates Rac-1 activation during cell entry by Leishmania amazonensis. Infect. Immun..

[87] Taoufiq Z., Gay F., Balvanyos J., Ciceron L., Tefit M., Lechat P., Mazier D. (2008). Rho kinase inhibition in severe malaria: Thwarting parasite-induced collateral damage to endothelia. J. Infect. Dis..

[88] Seixas E., Ramalho J.S., Mota L.J., Barral D.C., Seabra M.C. (2012). Bacteria and protozoa differentially modulate the expression of Rab proteins. PLoS ONE.

[89] Seixas E., Escrevente C., Seabra M.C., Barral D.C. (2018). Rab GTPase regulation of bacteria and protozoa phagocytosis occurs through the modulation of phagocytic receptor surface expression. Sci. Rep..

[90] Ólafsson E.B., Barragan A. (2020). The unicellular eukaryotic parasite *Toxoplasma gondii* hijacks the migration machinery of mononuclear phagocytes to promote its dissemination. Biol. Cell.

[91] da Silva C.V., da Silva E.A., Cruz M.C., Chavrier P., Mortara R.A. (2009). ARF6, PI3-kinase and host cell actin cytoskeleton in *Toxoplasma gondii* cell invasion. Biochem. Biophys. Res. Commun..

[92] Huber S., Bär A., Epp S., Schmuckli-Maurer J., Eberhard N., Humbel B.M., Hemphill A., Woods K. (2020). Recruitment of Host Nuclear Pore Components to the Vicinity of Theileria Schizonts. Msphere.

[93] Gao Y., Dickerson J.B., Guo F., Zheng J., Zheng Y. (2004). Rational design and characterization of a Rac GTPase-specific small molecule inhibitor. Proc. Natl. Acad. Sci. USA.

[94] Lemichez E., Aktories K. (2013). Hijacking of Rho GTPases during bacterial infection. Exp. Cell Res..

[95] Shutes A., Onesto C., Picard V., Leblond B., Schweighoffer F., Der C.J. (2007). Specificity and mechanism of action of EHT 1864, a novel small molecule inhibitor of Rac family small GTPases. J. Biol. Chem..

[96] Cardama G.A., Comin M.J., Hornos L., Gonzalez N., Defelipe L., Turjanski A.G., Alonso D.F., Gomez D.E., Menna P.L. (2014). Preclinical development of novel Rac1-GEF signaling inhibitors using a rational design approach in highly aggressive breast cancer cell lines. Anticancer Agents Med. Chem..

[97] Kalfa T.A., Pushkaran S., Mohandas N., Hartwig J.H., Fowler V.M., Johnson J.F., Joiner C.H., Williams D.A., Zheng Y. (2006). Rac GTPases regulate the morphology and deformability of the erythrocyte cytoskeleton. Blood.

[98] López-Gómez A., Cano V., Moranta D., Morey P., García del Portillo F., Bengoechea J.A., Garmendia J. (2012). Host cell kinases, α5 and β1 integrins, and Rac1 signalling on the microtubule cytoskeleton are important for non-typable Haemophilus influenzae invasion of respiratory epithelial cells. Microbiology.

[99] Chen X.M., Keithly J.S., Paya C.V., LaRusso N.F. (2002). Cryptosporidiosis. N. Engl. J. Med..

[100] Clark D.P. (1999). New insights into human cryptosporidiosis. Clin. Microbiol. Rev..

[101] Dobbelaere D., Heussler V. (1999). Transformation of leukocytes by *Theileria parva* and *T. annulata*. Annu. Rev. Microbiol..

[102] McGuire K., Manuja A., Russell G.C., Springbett A., Craigmile S.C., Nichani A.K., Malhotra D.V., Glass E.J. (2004). Quantitative analysis of pro-inflammatory cytokine mRNA expression in *Theileria annulata*-infected cell lines derived from resistant and susceptible cattle. Vet. Immunol. Immunopathol..

[103] Haubert D., Gharib N., Rivero F., Wiegmann K., Hösel M., Krönke M., Kashkar H. (2007). PtdIns(4,5)P-restricted plasma membrane localization of FAN is involved in TNF-induced actin reorganization. EMBO J..

[104] Lemichez E., Flatau G., Bruzzone M., Boquet P., Gauthier M. (1997). Molecular localization of the *Escherichia coli* cytotoxic necrotizing factor CNF1 cell-binding and catalytic domains. Mol. Microbiol..

[105] Stenmark H. (2009). Rab GTPases as coordinators of vesicle traffic. Nat. Rev. Mol. Cell Biol..

[106] Yeo J.C., Wall A.A., Luo L., Stow J.L. (2016). Sequential recruitment of Rab GTPases during early stages of phagocytosis. Cell. Logist..

[107] Karnoub A.E., Weinberg R.A. (2008). Ras oncogenes: Split personalities. Nat. Rev. Mol. Cell Biol..

[108] Donaldson J.G., Jackson C.L. (2011). ARF family G proteins and their regulators: Roles in membrane transport, development and disease. Nat. Rev. Mol. Cell Biol..

[109] Sztul E., Chen P.W., Casanova J.E., Cherfils J., Dacks J.B., Lambright D.G., Lee F.S., Randazzo P.A., Santy L.C., Schürmann A. (2019). ARF GTPases and their GEFs and GAPs: Concepts and challenges. Mol. Biol. Cell.

[110] Donaldson J.G. (2003). Multiple roles for Arf6: Sorting, structuring, and signaling at the plasma membrane. J. Biol. Chem..

[111] Boudhraa Z., Carmona E., Provencher D., Mes-Masson A.M. (2020). Ran GTPase: A Key Player in Tumor Progression and Metastasis. Front. Cell Dev. Biol..

[112] Kessel R.G. (1992). Annulate lamellae: A last frontier in cellular organelles. Int. Rev. Cytol..

[113] Huber S., Karagenc T., Ritler D., Rottenberg S., Woods K. (2018). Identification and characterisation of a *Theileria annulata* proline-rich microtubule and SH3 domain-interacting protein (TaMISHIP) that forms a complex with CLASP1, EB1, and CD2AP at the schizont surface. Cell. Microbiol..

[114] Antony H.A., Parija S.C. (2016). Antimalarial drug resistance: An overview. Trop. Parasitol..

[115] Fairhurst R.M., Dondorp A.M. (2016). Artemisinin-Resistant *Plasmodium falciparum* Malaria. Microbiol. Spectr..

[116] Montazeri M., Mehrzadi S., Sharif M., Sarvi S., Tanzifi A., Aghayan S.A., Daryani A. (2018). Drug Resistance in. Front. Microbiol..

[117] Zhu G., Yin J., Cuny G. (2021). Current status and challenges in drug discovery against the globally important zoonotic cryptosporidiosis. Anim. Dis..

[118] Prudêncio M., Mota M.M. (2013). Targeting host factors to circumvent anti-malarial drug resistance. Curr. Pharm. Des..

[119] Chiang C.Y., Uzoma I., Moore R.T., Gilbert M., Duplantier A.J., Panchal R.G. (2018). Mitigating the Impact of Antibacterial Drug Resistance through Host-Directed Therapies: Current Progress, Outlook, and Challenges. MBio.

[120] Stanley S.A., Barczak A.K., Silvis M.R., Luo S.S., Sogi K., Vokes M., Bray M.A., Carpenter A.E., Moore C.B., Siddiqi N. (2014). Identification of host-targeted small molecules that restrict intracellular Mycobacterium tuberculosis growth. PLoS Pathog..

[121] Guo L., Chen W., Zhu H., Chen Y., Wan X., Yang N., Xu S., Yu C., Chen L. (2014). Helicobacter pylori induces increased expression of the vitamin d receptor in immune responses. Helicobacter.

[122] Ottosen S., Parsley T.B., Yang L., Zeh K., van Doorn L.J., van der Veer E., Raney A.K., Hodges M.R., Patick A.K. (2015). In vitro antiviral activity and preclinical and clinical resistance profile of miravirsen, a novel anti-hepatitis C virus therapeutic targeting the human factor miR-122. Antimicrob Agents Chemother..

[123] de Wispelaere M., LaCroix A.J., Yang P.L. (2013). The small molecules AZD0530 and dasatinib inhibit dengue virus RNA replication via Fyn kinase. J. Virol..

[124] Crouchet E., Wrensch F., Schuster C., Zeisel M.B., Baumert T.F. (2018). Host-targeting therapies for hepatitis C virus infection: Current developments and future applications. Ther. Adv. Gastroenterol..

[125] Dorr P., Westby M., Dobbs S., Griffin P., Irvine B., Macartney M., Mori J., Rickett G., Smith-Burchnell C., Napier C. (2005). Maraviroc (UK-427,857), a potent, orally bioavailable, and selective small-molecule inhibitor of chemokine receptor CCR5 with broad-spectrum anti-human immunodeficiency virus type 1 activity. Antimicrob. Agents Chemother..

[126] Glennon E.K.K., Dankwa S., Smith J.D., Kaushansky A. (2018). Opportunities for Host-targeted Therapies for Malaria. Trends Parasitol..

[127] Szewczyk-Golec K., Pawłowska M., Wesołowski R., Wróblewski M., Mila-Kierzenkowska C. (2021). Oxidative Stress as a Possible Target in the Treatment of Toxoplasmosis: Perspectives and Ambiguities. Int. J. Mol. Sci..

[128] Brizuela M., Huang H.M., Smith C., Burgio G., Foote S.J., McMorran B.J. (2014). Treatment of erythrocytes with the 2-cys peroxiredoxin inhibitor, Conoidin A, prevents the growth of *Plasmodium falciparum* and enhances parasite sensitivity to chloroquine. PLoS ONE.

[129] Smith C.M., Jerkovic A., Puy H., Winship I., Deybach J.C., Gouya L., van Dooren G., Goodman C.D., Sturm A., Manceau H. (2015). Red cells from ferrochelatase-deficient erythropoietic protoporphyria patients are resistant to growth of malarial parasites. Blood.

[130] Hamie M., Najm R., Deleuze-Masquefa C., Bonnet P.A., Dubremetz J.F., El Sabban M., El Hajj H. (2021). Imiquimod Targets Toxoplasmosis through Modulating Host Toll-Like Receptor-MyD88 Signaling. Front. Immunol..

[131] Castillo-Pichardo L., Humphries-Bickley T., De La Parra C., Forestier-Roman I., Martinez-Ferrer M., Hernandez E., Vlaar C., Ferrer-Acosta Y., Washington A.V., Cubano L.A. (2014). The Rac Inhibitor EHop-016 Inhibits Mammary Tumor Growth and Metastasis in a Nude Mouse Model. Transl. Oncol..

[132] Humphries-Bickley T., Castillo-Pichardo L., Corujo-Carro F., Duconge J., Hernandez-O’Farrill E., Vlaar C., Rodriguez-Orengo J.F., Cubano L., Dharmawardhane S. (2015). Pharmacokinetics of Rac inhibitor EHop-016 in mice by ultra-performance liquid chromatography tandem mass spectrometry. J. Chromatogr. B Analyt. Technol. Biomed. Life Sci..

[133] Prieto-Dominguez N., Parnell C., Teng Y. (2019). Drugging the Small GTPase Pathways in Cancer Treatment: Promises and Challenges. Cells.

[134] Bid H.K., Roberts R.D., Manchanda P.K., Houghton P.J. (2013). RAC1: An emerging therapeutic option for targeting cancer angiogenesis and metastasis. Mol. Cancer Ther..

[135] Genetu Bayih A., Debnath A., Mitre E., Huston C.D., Laleu B., Leroy D., Blasco B., Campo B., Wells T.N.C., Willis P.A. (2017). Susceptibility Testing of Medically Important Parasites. Clin. Microbiol. Rev..

[136] Meneceur P., Bouldouyre M.A., Aubert D., Villena I., Menotti J., Sauvage V., Garin J.F., Derouin F. (2008). In vitro susceptibility of various genotypic strains of *Toxoplasma gondii* to pyrimethamine, sulfadiazine, and atovaquone. Antimicrob. Agents Chemother..

[137] Makler M.T., Ries J.M., Williams J.A., Bancroft J.E., Piper R.C., Gibbins B.L., Hinrichs D.J. (1993). Parasite lactate dehydrogenase as an assay for *Plasmodium falciparum* drug sensitivity. Am. J. Trop. Med. Hyg..

[138] Konreddy A.K., Rani G.U., Lee K., Choi Y. (2019). Recent Drug-Repurposing-Driven Advances in the Discovery of Novel Antibiotics. Curr. Med. Chem..

[139] Trivedi J., Mohan M., Byrareddy S.N. (2020). Drug Repurposing Approaches to Combating Viral Infections. J. Clin. Med..

[140] Tiede I., Fritz G., Strand S., Poppe D., Dvorsky R., Strand D., Lehr H.A., Wirtz S., Becker C., Atreya R. (2003). CD28-dependent Rac1 activation is the molecular target of azathioprine in primary human CD4+ T lymphocytes. J. Clin. Invest..

[141] Oprea T.I., Sklar L.A., Agola J.O., Guo Y., Silberberg M., Roxby J., Vestling A., Romero E., Surviladze Z., Murray-Krezan C. (2015). Novel Activities of Select NSAID R-Enantiomers against Rac1 and Cdc42 GTPases. PLoS ONE.

[142] Bobbala D., Koka S., Geiger C., Föller M., Huber S.M., Lang F. (2009). Azathioprine favourably influences the course of malaria. Malar. J..

